# The Revolution in the after Treatment of Cataract Operations

**Published:** 1887-11

**Authors:** Julian J. Chisholm

**Affiliations:** Surgeon in charge of the Presbyterian Eye and Ear Charity Hospital, of Baltimore, Md.


### THE REVOLUTION IN THE AFTER TREATMENT OF CATARACT OPERATIONS.

By JULIAN J. CHISHOLM, M. D.,
**Surgeon in charge of the Presbyterian Eye and Ear Charity Hospital, of Baltimore, Md.**

At the meeting of the American Medical Association, in May, 1886, at St. Louis, I brought before the ophthalmological section a statement from Dr. Charles Michel, of St. Louis, that he was treating successfully his cataract extraction cases with eyes closed by adhesive strips in moderately lighted rooms. The section disapproved the plan, and advised a continuance of the method in universal use of compress bandages and dark rooms. I informed the section that I would put the proposed method on trial, and would report to the section next year, in Chicago, the results of my experiments. That report has been rendered. During the year, ninety-eight cataract extractions and sixty-nine iridectomies have been performed, with such a percentage of successes as warrants the statement that bandages and dark rooms are not only permanently abandoned at the hospital, but must in the very near future be given up by all ophthalmic surgeons.

During the course of this year's experiments, not only have light isinglass superseded the heavy compresses and bandages, but m uch of the restraint in universal use has been proved useless and arbitrary. Under the belief that when the two lids are made one by the adhesive strap, with the tarsal cartilages acting as splints over the corneal surface, and kept in position by means of the tonic contraction of the palpebral muscle, the eye recently operated upon was thoroughly protected from disturbances, regardless of the movements of the rest of the body, the many restraints in universal practice were one by one abandoned.

First, it was found not necessary to operate in the bed in which the patient was to remain during the after treatment. For this was substituted an operating table of convenient height and width, placed near a large window, from which good light could be had. This permits the operator to complete the manual to his own satisfaction. When the operation for cataract extraction is smoothly done, nine-tenths of the dangers against the restoration of sight are removed.

The next step in the abandoning of restraints was not to put the patients to bed, but to allow them the use of their limbs during the entire treatment. Dr. Michel, the advocate of the light room and light dressing treatment, still adopts the restraints in common use, not allowing his patients to turn from their backs for five days. When at the end of this period their backs were aching, he would allow them to turn over on the side opposite to the eye operated upon. I do not put them to bed at all. After the operation I allow them to use a lounge, bed, or chair, following their own preferences. They go to bed at their usual bedtime hour, sleeping on any side that it is most comfortable, and they dress themselves in the morning before breakfast.

For the past four months I have taken another great step forward, and have released the eye not operated upon from being strapped. This was a bold step in defiance of the theory universally accepted, that the movements of the eye, when left open, must affect the cornea of the other recently cut, and, therefore, a needful rest must be secured. However satisfying this theory may be—and we have all adopted it for generations back—the experience of the Presbyterian Eye and Ear Hospital, of Baltimore, has conclusively shown that this restraint was never called for and had never been of any utility, but, on the contrary, of much annoyance and discomfort.

Thus one by one the old rules of universal adoption have been abandoned, and I may say that now the revolution in the after treatment of cataract cases has become complete.

Hereafter there will be no more bandaging, dark rooms, bed operations, bed restraints, diet lists, isolation, or smoked glasses needed. The year's work at the hospital has shown that the red, suffused, watery, sensitive eyes, so constantly seen after cataract operations, were made so by the restraining treatment, and were not necessary accompaniments of the convalescence. Thick bandages, dark rooms, and restraint in bed caused most of these annoyances. When cataract extractions are treated with a very thin, light-colored isinglass strip over one eye as the sole dressing, leaving the other eye open, the patient allowed to enjoy in his chamber the light to which he is daily accustomed, the strap removed at the end of the fifth day, when the corneal wound is perfectly healed, very little sensitiveness or congestion or watering will be found. Convalescence is in this way very much expedited. At the end of the first week the patient can be allowed the privilege of the entire house, and before the two weeks are finished he will be ready for dismissal, with eyes so strong as to need but little protection from smoked glass.

The fruits of my early experiments were given to the profession in June last, with reasons for the change in treatment. These were deemed satisfactory by many specialists, who, upon my recommendation, determined to try the new plan for themselves. At the Chicago meeting, June 7, 1887, many were found in the section who were as enthusiastic as myself over the new after treatment. Several had used the isinglass strap and light rooms, and expressed themselves as delighted at the beautiful results secured. At my suggestion they have promised to test equally the no bed treatment, leaving one eye open for the guidance of the patient, so as really to remove all restraint. This is to be the dressing of the future, and is an immense advance over the blind groping of both patient and surgeon as now conducted.

My present improved practice is to treat the wound made in the extraction of cataract as if it were an ordinary corneal wound, such as we daily see resulting from accident. Close the eye with a piece of isinglass plaster, and restrict the patient to his chamber for a few days.

What a change is this over the course still adhered to by some as expressed at the Chicago meeting! First preparation of patient, then operating in the bed, the carefully and thoroughly excluding light from both eyes with compresses and head bandages, in a dark room, then restraint in bed, patients not allowed to talk to friends or to eat solid food, must stay on their backs, even with hands tied to prevent an accidental touching of the eyes while asleep, and this cruelty kept up for days in the name of progressive surgery. To be sure such statements only came from old practitioners, who had been so long running in this deep rut that they could not get out of it, and yet up to one year since this was orthodox practice, sanctioned by every authority on cataract operations.

The work of the Presbyterian Eye and Ear Charity Hospital for the past year has broken the spell, and a number of specialists who have tested successfully the new plan have renounced altogether the old method. From present appearances, it would look as if the dark room and confining after treatment of the cataract and iridectomy cases will soon be assigned to the shelves of a surgical museum, and all such patients will be allowed to enjoy the blessed light of day throughout their entire treatment, for their own immediate benefit and also for the comfort of the attendants.

